# ZnO Nanowires/N719 Dye With Different Aspect Ratio as a Possible Photoelectrode for Dye-Sensitized Solar Cells

**DOI:** 10.3389/fchem.2020.604092

**Published:** 2021-02-02

**Authors:** Karina Portillo-Cortez, Ana Martínez, Monserrat Bizarro, Mario F. García-Sánchez, Frank Güell, Ateet Dutt, Guillermo Santana

**Affiliations:** ^1^Departamento de Materiales de Baja Dimensionalidad, Instituto de Investigaciones en Materiales, Universidad Nacional Autónoma de México, Ciudad de México, Mexico; ^2^Unidad Profesional Interdisciplinaria en Ingeniería y Tecnologías Avanzadas, Instituto Politécnico Nacional, Ciudad de México, Mexico; ^3^ENFOCAT-IN2UB, Universitat de Barcelona, Barcelona, Spain

**Keywords:** ZnO nanowires, high aspect ratio, dye-sensitized solar cell, N719 dye, Au nanoparticles, absorption spectra

## Abstract

The vapor-liquid-solid (VLS) process was applied to fabricate zinc oxide nanowires (ZnO NWs) with a different aspect ratio (AR), morphological, and optical properties. The ZnO NWs were grown on a system that contains a quartz substrate with transparent conductive oxide (TCO) thin film followed by an Al-doped ZnO (AZO) seed layer; both films were grown by magnetron sputtering at room temperature. It was found that the ZnO NWs presented high crystalline quality and vertical orientation from different structural and morphological characterizations. Also, NWs showed a good density distribution of 69 NWs/μm^2^ with a different AR that offers their capability to be used as possible photoelectrode (anode) in potential future device applications. The samples optical properties were studied using various techniques such as photoluminescence (PL), absorption, and transmittance before and after sensitization with N719 dye. The results demonstrated that NW with 30 nm diameter had the best characteristics as feasible photoelectrode (anode) (high absorption, minimum recombination, high crystallinity). Also, the present samples optical properties were found to be improved due to the existence of N719 dye and Au nanoparticles on the tip of NWs. NWs grown in this work can be used in different photonic and optoelectronic applications.

## Introduction

Energy production is satisfied by conventional energetic technologies based on natural gas, coal, oil. Several efforts have been performed to mollify the growing demand for electrical energy in unification with ecological upkeep through renewable energy development (Abdin et al., 2013; Skandalos and Karamanis, 2015; Greenaway et al., 2017; Pathak, 2020). O'Regan and Grätzel in 1991 proposed a modern kind of renewable solar technology termed as Dye-sensitized solar cell (DSSC) (O'Regan and Grätzel, 1991) that convert solar irradiation to electricity via a similar process to photosynthesis. In a DSSC device, a dye sensitizer such as N719 dye is one of the most critical parts where the majority of the absorption of incident solar radiation occurs to generate electrons that are injected and transported by an Electron Transport Layer (ETL), and finally, collected by a TCO substrate (Portillo-Cortez et al., 2019). The electrons flow through the external circuit toward the counter electrode. The regeneration of the oxidized dye is due to a redox reaction produced inside the electrolyte (Boschloo, 2019). Outstanding their simplicity in the fabrication technique and economical way of processing, these methods have drawn a significant consideration as in contrast to traditional solar cells (Bisquert et al., 2004; Grätzel, 2005; Wang et al., 2006; Gong et al., 2012, 2017; Jung and Lee, 2013; Scalia et al., 2018; Juang et al., 2019) and also present satisfactory conversion proficiency (13 –14%) (Hagfeldt et al., 2010; Parisi et al., 2014; Freitag et al., 2017). As mentioned earlier, optimizing the structure with maximum absorption and least electron-hole recombination is one of the significant challenges in designing and manufacturing of photoelectrodes for their use in the DSSC device. It is also important to mention that the short circuit current and open-circuit voltage of the device is strongly dependent on the overall absorption and recombination processes, which is one of the need of the hour for the fabrication of these devices. One of the most common semiconductors is TiO_2_, which is widely used as a photoelectrode (anode) in a DSSC device due to the great surface area of the network nanoparticles and physical and chemical stability; however, ZnO is an exciting alternative for the electrode due to the similar, even better electrical properties compared to TiO_2_ (Canto-Aguilar et al., 2017; Boschloo, 2019).

ZnO is a semiconductor of the II-VI group. It presents a broad direct bandgap of 3.3–3.37 eV at 300 K and a large exciton binding energy of 60 meV (Galdamez et al., 2019); also, it has higher electron mobility (130–200 cm^2^V^−1^s^−1^) and diffusion coefficient (1.1^*^10^−4^ cm^2^s^−1^). ZnO shows three crystallographic phases: hexagonal wurtzite, zinc blend, and rock salt, and can be synthesized with a varied choice of morphologies such as nanoparticles, nanorods, nanowires, nanobelts, nanoflowers, hierarchical and core-shell structures (Zhang et al., 2012; Yang et al., 2015). Among all ZnO nanostructures, ZnO nanowires (ZnO NWs) are of the main interest due to their high aspect ratio (AR), the proportion of length/diameter and is related to the effective surface area. Also, ZnO NWs present effective confinement of photons and carriers (Song et al., 2010; Cui, 2012), and they provide a direct and fast pathway to the transportation of free charge carriers (Karst et al., 2011; Lee et al., 2011; Meng et al., 2014). These previous properties suggest ZnO NWs as a good candidate for UV laser, photocatalysis, solar cells, LEDs, and other photonic applications (Hong et al., 2008; Kang et al., 2008; Wijeratne and Bandara, 2014; Polyakov et al., 2016).

The manufacturing techniques of ZnO NWs can primarily be categorized as vapor and solution phase synthesis (physical and chemical) (Cui, 2012; Udom et al., 2013). Vapor-liquid-solid (VLS) is a kind of vapor-phase synthesis (physical) for ZnO NW. VLS process is widely used to obtain nanostructures with controlled features: dimensions, shapes, distribution, nanostructure density, even high crystalline quality. These useful structural and morphological features can provide excellent charge transport properties for photovoltaic and nanoelectronics (Yang et al., 2008; Simon et al., 2013; Ye et al., 2015). The VLS process involves using liquid metal nanoparticles as a catalyst (Au), and it constructs a eutectic alloy with the seed layer and gas precursor. Then, the gas precursor dissolves into the liquid catalyst to form a supersaturated solution followed by the nucleation process and NWs growth (Güell and Martínez-Alanis, 2019). For ZnO, the dimensions and quality of NWs are dependent on the different VLS factors such as chamber pressure, oxygen ratio, and thickness of the metal catalyst layer (Zhang et al., 2012).

In the previous investigations, it has been shown that 1-D ZnO nanostructures fabricated by different chemical methods (Gao et al., 2007) can be used as photoelectrodes in DSSC solar cells. Still, there were remaining some open questions, for example, the effect of the following factors, such as (1) Quality of crystallinity and Optimum morphology (diameter and length) of NWs on the performance of a DSSC device. (2) Repeatability and control over the fabrication of NWs. (3) Better surface area for the overall enhancement in the efficiency of the device (Saleem et al., 2019). One of the disadvantages of ZnO NWs for potential application as photoelectrode could be low chemical stability when immersed in the acid environment generated by the dye dissolution. Fortunately, many proposals have been performed to overcome the chemical stability problem. These proposals have improved the DSSC device performance (Sakai et al., 2013; Warnan et al., 2013).

In early works, our research group has demonstrated broad control over the growth of ZnO NWs through the VLS method, where the effect of seed layer, vertical and random orientation of the NWs on the structural, morphological and optical properties have been analyzed. Moreover, we have reported the application of these ZnO NWs in bio-sensing and hydrogen production (Serrano et al., 2017; Galdamez et al., 2019; Galdámez-Martínez et al., 2020a). Additionally, in previous work, we have reported the theoretical study of the absorption spectra of N719 dye together with the ZnO NWs, and it was found that a particular N719 group showed the best alignment of the bands for the better absorption, charge-transport mechanism, and rate of regeneration (Portillo-Cortez et al., 2019). From that experience, we wanted to start implementing the fabrication of the first photoelectrode prototype based on ZnO NWs grown by the VLS process and their relationship with N719 dye for improved absorption.

In the present work, we show the fabrication of ZnO NWs with a different aspect ratio (AR) and good crystallinity obtained by the VLS process. Four different thicknesses of the Au layer are utilized to control the AR, shape, and density of NWs. The optical properties were studied before and after the sensitization with the N719 dye of the ZnO NWs. To the best of our knowledge, this is the first time that AR of ZnO NWs grown by VLS is optimized for potential application as photoelectrodes to be used in a DSSC device. The present research could lead to a suitable merge in modern organic solar cell technology such as Perovskites and other optoelectronic applications.

## Materials and Methods

### TCO and Seed Layer (SL) Deposition

Both TCO and SL films are composed of AZO and were deposited on quartz substrates (1.5 × 2.5 cm) by a magnetron sputtering system (mod. H2 INTERCOVAMEX®). A solid target of AZO (99.999 %, ZnO 98 wt %: Al_2_O_3_ wt %, Kurt Lesker®) was used as source material. The chamber pressure was maintained at 9.3 × 10^−5^ mbar, and a mass flow controller regulated the Ar gas (99.999%) flow rate. The sputtering process was performed similarly, as reported previously (Serrano et al., 2017). Deposition conditions for TCO were 45 W sputtering power, 5 sccm Ar flow for 12 min, and the substrate-target distance was 4 cm. Subsequently, the SL film was deposited at 45 W, 35 sccm Ar flow for 6 min, and the substrate-target distance was kept at 10 cm. All the sputtering growth processes were carried out at room temperature. After both deposition processes, the sample is labeled as TCO/SL.

### ZnO NW Photoelectrodes Structure by VLS

ZnO NW samples were prepared by the VLS method on TCO/SL substrates previously deposited for their use as a photoelectrode (TCO/SL/NWs). At this point, the TCO/SL samples were coated with a thin Au layer via the DC sputtering process with four different thicknesses (Table 1). The VLS process was performed in a tubular oven (HAT-1200, EVELSA®). For the carbothermal reduction, ZnO powder (99.999%) and graphite (99.99%) were placed in a quartz container, whereas the sample TCO/SL coated with the Au layer was placed in a second substrate holder. The quartz containers were placed inside the oven, followed by an annealing process at 950°C for 1 h under (3.3 L/min) Ar atmosphere.

**Table 1:** Morphological properties of the ZnO NWs in relation to different Au thicknesses.

**Sample**	**Au thickness (nm)**	**NW diameter (nm)**	**NW length (nm)**	**NW aspect ratio (AR)**
NW (*d* = 30 nm)	1	30	3,000	100
NW (*d* = 42 nm)	2	42	2,800	67
NW (*d* = 60 nm)	4	60	2,700	45
NW (*d* = 85 nm)	6	85	3,000	35

### Sensitization Process of ZnO NW

**Figure 1:**
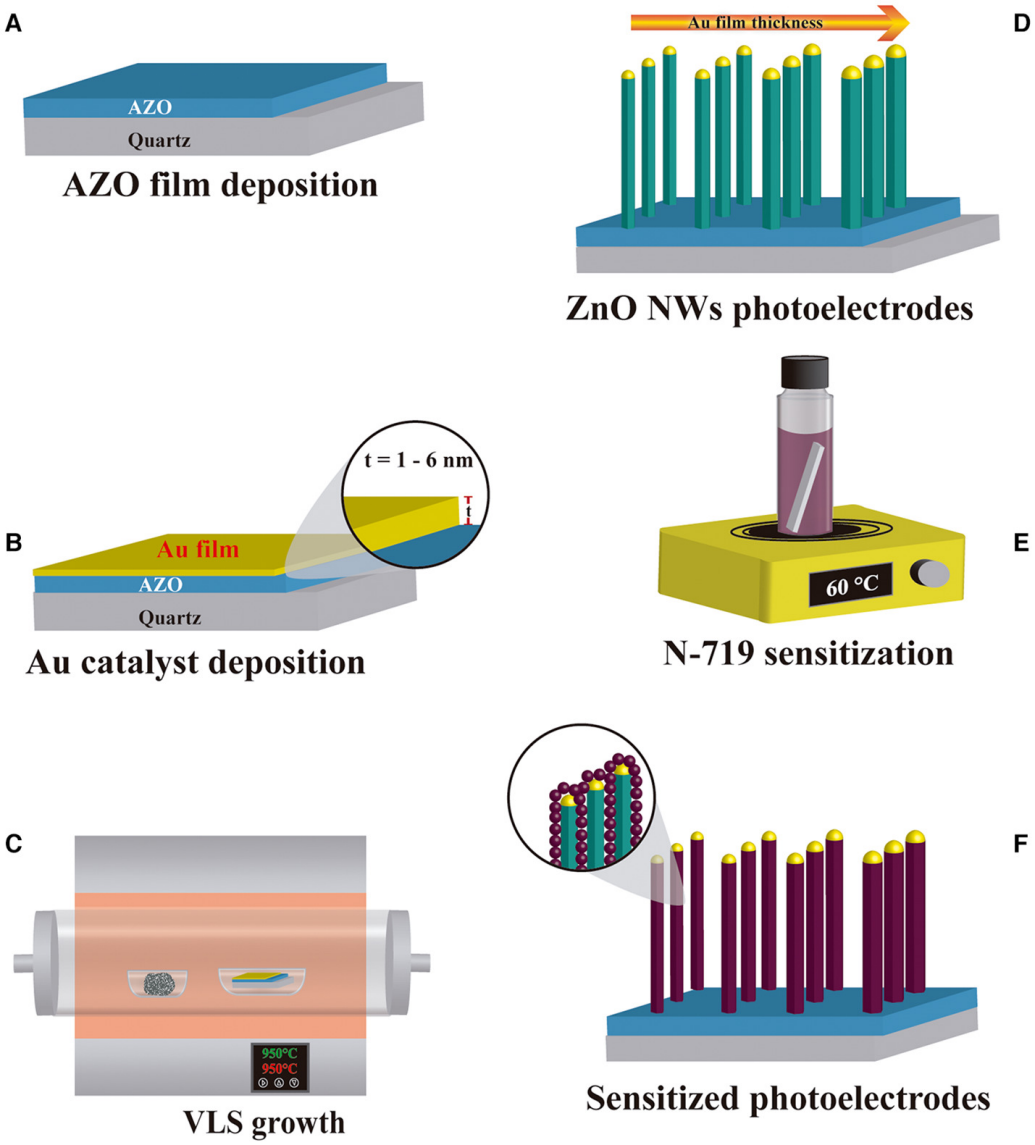
Elaboration process of ZnO NWs based DSSC photoelectrode prototype. **(A)** AZO and **(B)** Au film deposition, **(C)** VLS process, **(D)** ZnO NWs samples, **(E)** sensitization with N719 dye, and **(F)** final photoelectrodes.

The four ZnO NW photoelectrodes prototypes were sensitized with 0.5 mM of N719 dye (Ruthenizer 535-bisTBA from Solaronix®) in an ethanolic solution (Sigma Aldrich) at 60°C for 2 h. Following sensitization, the samples were extracted from the dye solution and meticulously cleaned with ethanol to remove the dye residues. The elaboration process of ZnO NW photoelectrode architecture is shown in Figure 1, which displays (A) AZO as a seed layer and (B) Au film deposition followed by (C) ZnO NWs growth by VLS and (D) obtention of ZnO NWs with different AR. (E,F) show the sensitization process of ZnO NWs with N719 dye.

### Characterization

The structural characterization of the samples was obtained by XRD (Bruker D8) with Cu-Kα radiation of 1.54 Å, 2θ angle from 25° to 65°. Morphological properties were analyzed via Field emission scanning electron microscopy (FESEM) in a JEOL JSM-7600F. JEOL ARM 200F was used to carry out the Transmission electron microscopy (TEM) measurements. Optical properties were studied using a Cary-5000 UV-Vis-NIR spectrophotometer with an integrating sphere in the range from 250 to 950 nm. Kimmon Koha He-Cd laser was used for the photoluminescence measurements.

## Results and Discussion

The four ZnO NWs samples presented different AR, as listed in Table 1. The variation in AR is due to the four thicknesses of Au layers deposited as a metal catalyst for the VLS process, that resulted in a change of the diameter of the Au liquid drops and, as a consequence, different diameters of the nanowires are obtained (Güell et al., 2016). Table 1 shows that the diameter of NWs augmented with increasing the Au layer thickness, whereas the length was 3 μm approximately for all samples (Please refer to SEM Micrographs for graphical details and histogram descriptions, **Figure 4**). The AR increased with a decrease in the diameter of the NWs, and the maximum AR was obtained with a diameter of 30 nm, which states the reverse relationship between the AR and the diameter of NWs. It is essential to mention that VLS offers this advantage of obtaining thin NWs compared to conventional chemical synthesis methods. This result is significant because a high value of AR is related to a high surface area in order to perform a superficial process that subsequently could be used for different applications such as gas sensors or catalysis.

**Figure 2:**
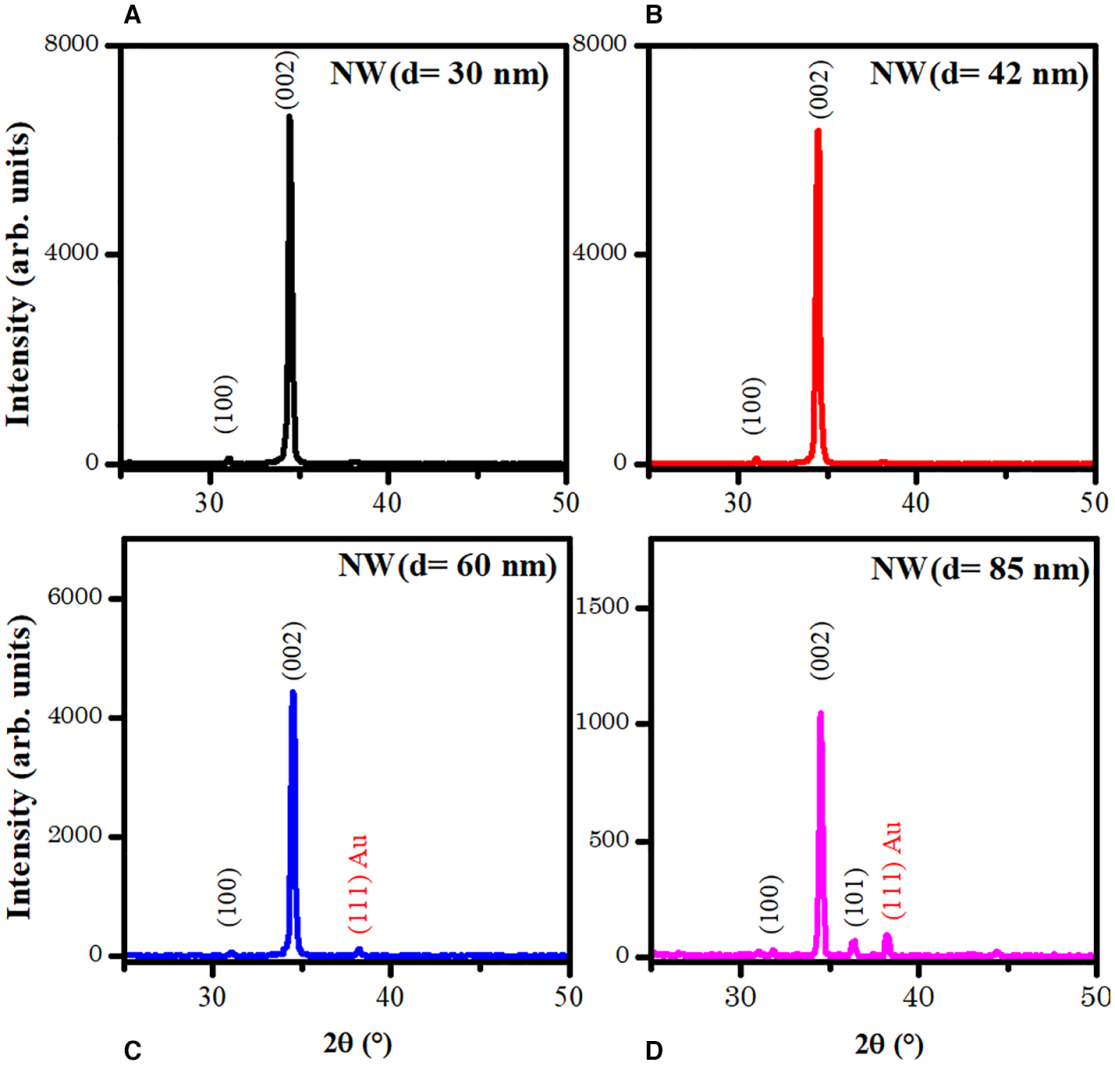
XRD patterns of the four ZnO NW samples with different AR. **(A)** NW (*d* = 30 nm), **(B)** NW (*d* = 42 nm), **(C)** NW (*d* = 60 nm) and **(D)** NW (*d* = 85 nm).

Figure 2 presents the XRD patterns of the NWs samples with different AR. The patterns show the peaks corresponding to (100), (002), and (101) planes of ZnO, and (111) plane of Au phases according to database sheets ICDD 01-070-2551 and ICDD 03-065-2870 for ZnO and Au, respectively. No secondary phases were identified for aluminum compounds from AZO layers. Also, the XRD peaks intensity shows a decrease when AR decreased from 100 to 35 (*d* = 30 nm to *d* = 85 nm, Table 1). The sample NW (*d* = 30 nm) showed the highest peak intensity, resulting in better crystal quality.

All patterns show a preferential crystallographic orientation along (002) peak, which corresponds to a c-orientation perpendicular to the substrate. The texture coefficient τ_(hkl)_ was determined by the followed expression (Hsu et al., 2005; Karst et al., 2011):

(1) $$\begin{array}{r} \tau_{\left(hkl\right)}=\frac{\frac{I_{\left(hkl\right)}}{I_{o\left(hkl\right)}}}{\frac{1}{N}\sum_{N}\left(\frac{I_{\left(hkl\right)}}{I_{o\left(hkl\right)}}\right)} \end{array}$$

Where *I*_(*hkl*)_ corresponds to the noticed intensity of the (hkl) plane, *I*_*o*(*hkl*)_ corresponds to the standard intensity of (hkl) plane from the database sheet, and N is the number of the considered diffraction peaks, *N* =3 for this work. Table 2 shows the texture coefficient for (100), (200), and (101) planes; all the samples have a preferred orientation along the c-axis due to the seed layer. However, the crystallite size showed no trend.

**Table 2:** Crystallite size and textured coefficient of ZnO NW photoelectrodes.

**Sample**	**Crystallite size (nm)**	**τ_(100)_**	**τ_(002)_**	**τ_(101)_**
NW (*d* = 30 nm)	35.8	0.09	2.97	0.01
NW (*d* = 42 nm)	33.6	0.03	2.97	0.01
NW (*d* = 60 nm)	34.7	0.03	2.97	0.01
NW (*d* = 85 nm)	36.2	0.04	2.90	0.06

**Figure 3:**
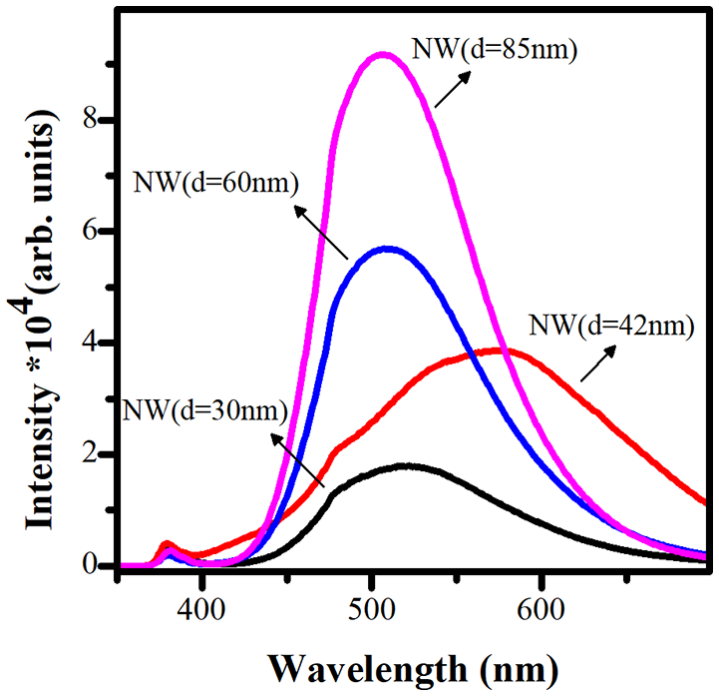
PL spectra of the four ZnO NW photoelectrodes with different AR.

The photoluminescence spectroscopy (Figure 3) was used as a cross-check technique to examine the crystalline quality and defect states of ZnO NWs in the present work. The PL relative intensities were found to be inversely related to the ZnO NWs diameter (Minimum for 30 nm, maximum for 85 nm). It is well known that the origin of the intense band in the visible region of the ZnO NWs is due to different defect states on the surface and inside of the structures. More discussions about the various luminescence mechanism centers from ZnO NWs can be found in our recent review article (Galdámez-Martinez et al., 2020b). As shown in Figure 3, the luminescence intensity decreases with the diminution of the NW diameter or increase in the AR. This is also related to a minimal point defect density and better crystallinity of the nanostructures, as shown in the XRD patterns (Figure 2). On the other hand, it is well known that radiative recombination, such as photoluminescence, is a kind of loss mechanism, and for solar cell photoelectrode applications, it is crucial not to have them. It can also be seen that the minimum PL intensity in the case of NWs with 30 nm diameter is the suitable sample as a photoelectrode (anode) in a possible DSSC device. On the other hand, broad NWs with a diameter such as 85 nm and intense PL can be used for different photonic or optoelectronic applications.

**Figure 4:**
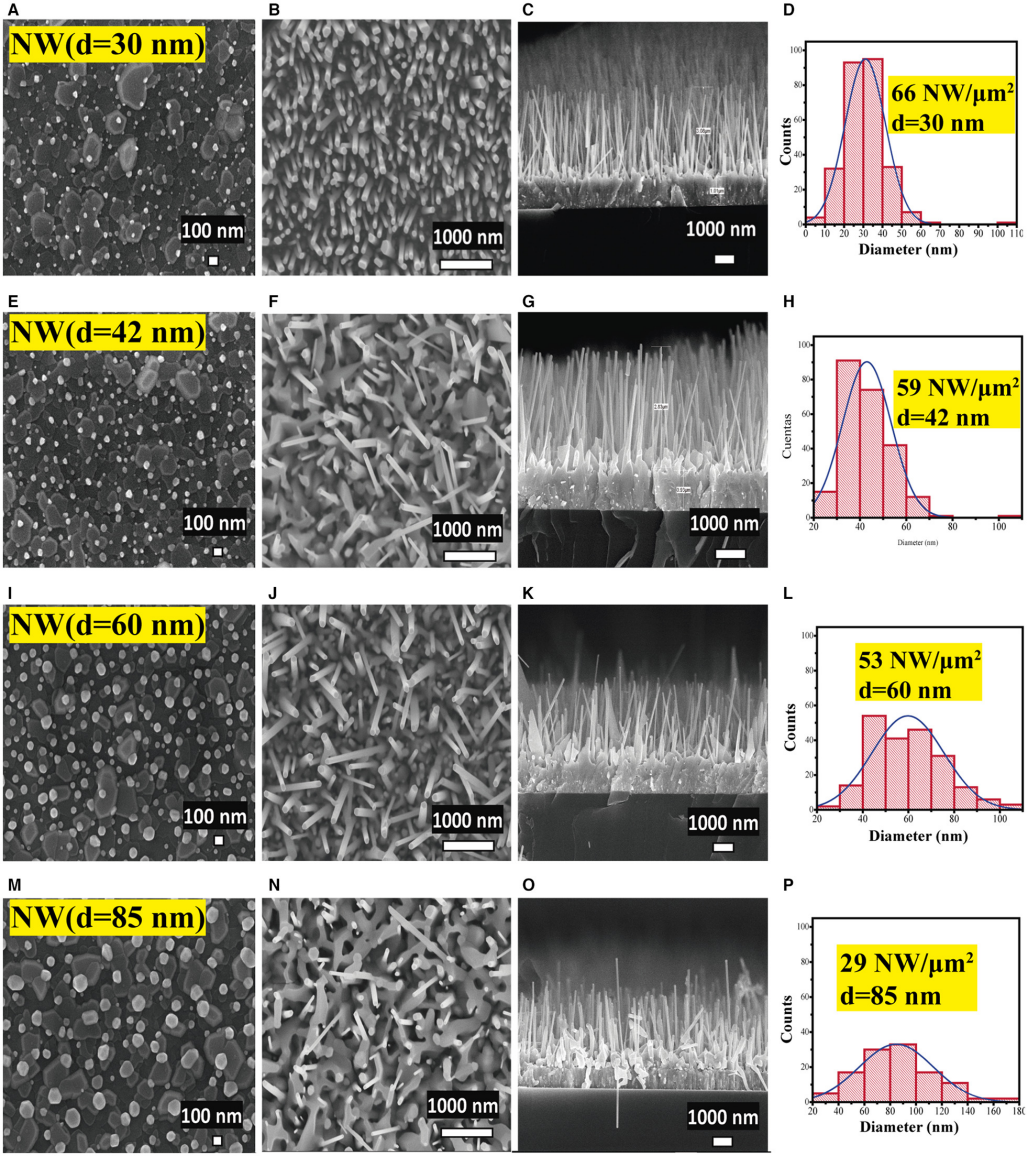
SEM images of **(A,E,I,M)** gold drop distribution and, **(B,F,J,N)** planar view and **(C,G,K,O)** cross-section view images, and **(D,H,L,P)** diameter histogram of ZnO NW photoelectrodes with different AR.

Figure 4 shows the morphological analysis of ZnO NWs with different AR. Figures 4A,E,I,M display the SEM micrographs of the Au drops distribution formed after thermal treatment at 950°C on the AZO substrates coated with different thicknesses of Au layers as listed in Table 1. As can be seen, the Au drop size was found to be enhanced (Figure 4) with the growing thickness of the Au layer (Table 1). Figures 4B,F,J,N show a planar view of the ZnO NWs with homogeneous distribution, which showed a decrement in the density related to the increase in NW diameter. The cross-sectional SEM images of the samples (TCO/SL/NWs) are displayed in Figures 4C,G,K,O. The ZnO NWs present perpendicular growth with respect to the substrate; this result is in good agreement with c-axes orientation obtained in the XRD study, Figure 2. The average length of the NWs was close to 3 μm (Table 1) for all the samples. This result could be due to the fact, in the VLS process, the length depends on the source powder quantity (0.2 g) and the VLS process time (1 h), which remained constant for all of the samples in this work.

Finally, Figures 4D,H,L,P present the diameter distribution, and surface density (histograms) of NWs obtained for each photoelectrode prototype calculated from Figures 4B,F,J,N. The diameter size of the NWs increased from 30 to 85 nm. In contrast, its uniformity decreased with the diameter increase, as can be seen from the histograms. A high surface density of NWs of 66 NW/μm^2^ was achieved by NW (*d* = 30 nm) sample, which decreased with an increase in diameter until 29 NW/μm^2^ (*d* = 85 nm). These results again confirm NW (*d* = 30 nm) as a suitable sample for prospective photoelectrode applications based on ZnO NWs due to desirable properties such as high AR, uniformity, better crystallinity, and higher surface density of the NWs.

**Figure 5:**
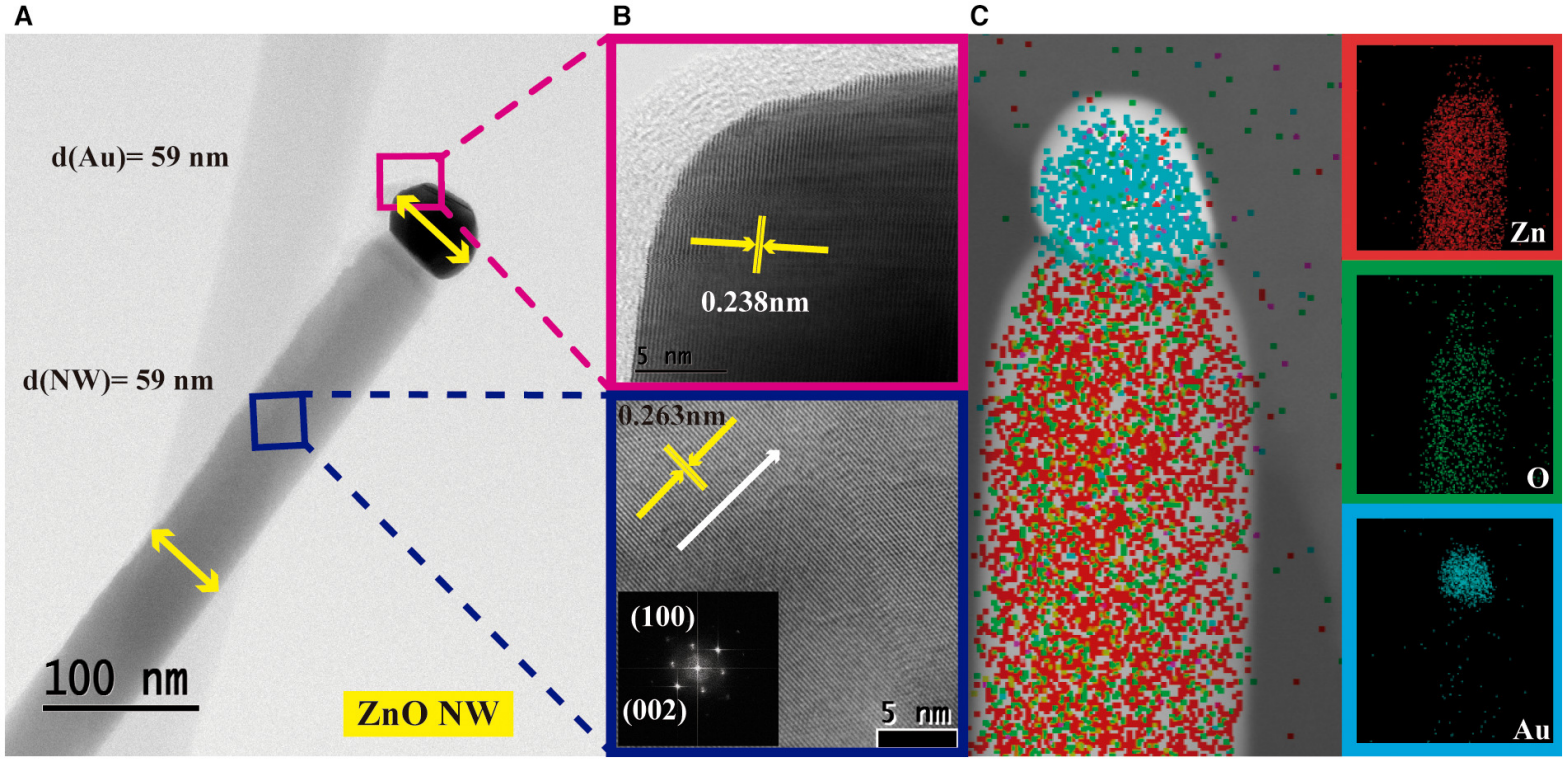
**(A)** TEM image, **(B)** HRTEM and FFT images and **(C)** EDS spectrum of single ZnO NW.

After obtaining a majority of the morphological information from the SEM study, additional analysis [TEM and STEM (Scanning Transmission Electron Microscopy)] was performed to cross-check the crystalline quality of the ZnO NWs grown by the VLS process and also the formation of Au on the tip of NWs. Figure 5A shows the micrograph of one of the deposited ZnO NW (*d* = 60 nm) to confirm the relationship between the Au diameter and the final diameter of the grown ZnO NW. Micrograph presents a clean and smooth longitudinal feature with Au drop placed on the NW tip that confirms VLS as the nanostructures growth mechanism. After statistical studies, the ZnO NWs average diameter was 59 nm, which is in good concordance with the diameter of the Au catalyst (59 nm). The similar diameter of both nanostructures supports the fact that the Au catalyst determines the diameter of the NWs obtained during the VLS process. Figure 5B displays the high-resolution TEM and Fourier-transformed pattern for the ZnO NW structure (blue) and the Au drop (pink). The ZnO NW shows high crystalline quality, and the determined interplanar spacing of 0.263 nm concurs with that of the (002) plane in c-axes orientation for wurtzite hexagonal ZnO. The lattice spacing for Au was estimated at 0.238 nm. Figure 5C presents STEM elemental mapping of the ZnO NW where Zn (red) and O (green) can be identified homogeneously along the NW, whereas Au (cyan) is localized on the tip of the NW.

**Figure 6:**
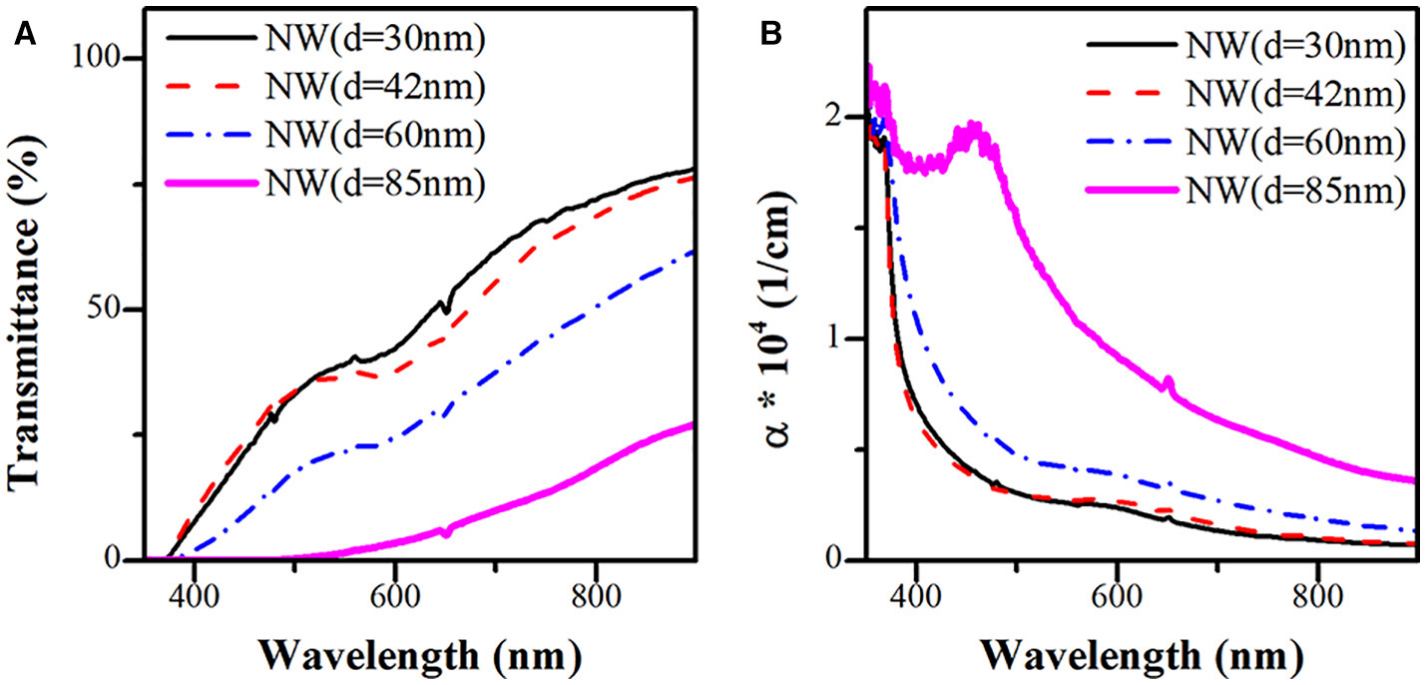
**(A)** Transmittance and **(B)** absorption coefficient of ZnO NW photoelectrodes.

Figures 6A,B present the transmittance spectrum and calculated effective absorption coefficient of the four ZnO NWs samples (photoelectrode prototype) with different AR. From Figure 6A, no transmittance signal is observed in the UV region of the electromagnetic spectrum due to the intrinsic absorbance properties of ZnO. Nevertheless, the transmittance increases at a longer wavelength. In the visible region, the transmittance signal increases with increasing the AR (decrease of the diameter) of the ZnO NWs. The peaks located in the visible region (450–600 nm) are associated with surface plasmon resonance (SPR) due to Au nanoparticles localized in the tip of ZnO NWs. Figure 6B displays the absorption coefficient of the NWs (α), which can be determined by the formula shown below (Bedia et al., 2015):

(2) $$\begin{array}{r} \alpha =\frac{1}{d}\text{ln}\left(\frac{100}{T}\right) \end{array}$$

where *d* corresponds to the thickness of the NWs, and *T* is the transmittance. As can be seen, α decrease with increasing both the wavelength and the AR of the ZnO NWs.

**Figure 7:**
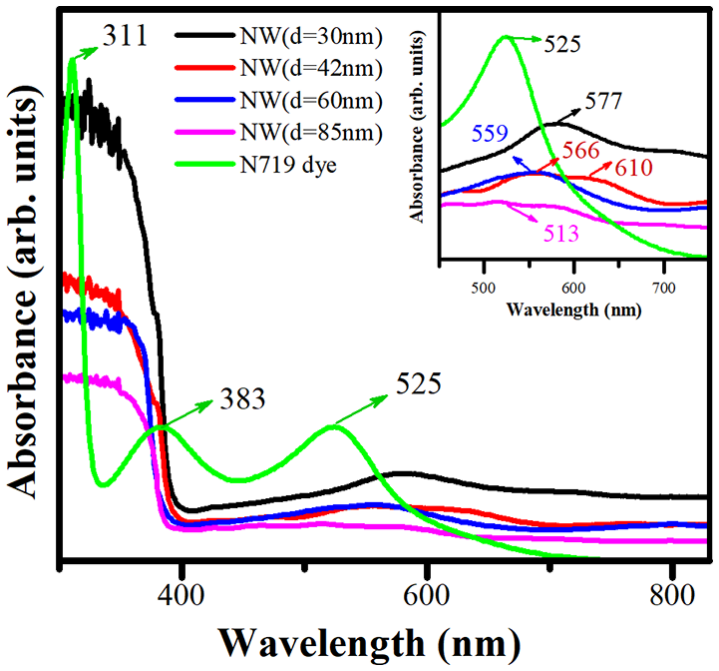
Absorbance spectra of ZnO NW photoelectrodes with different AR and N719 dye. The insert displays a magnification in the 450–700 nm region.

Figure 7 shows the experimental absorbance spectra measured by the Cary-5000 UV-Vis-NIR spectrophotometer with an integrating sphere of N719 dye and ZnO NW photoelectrodes with different AR without sensitization. For N719 dye, two absorption bands were observed in the UV region (311 and 383 nm), and one absorption band was identified in the visible region (525 nm). The absorption signal of the N719 dye is extended to 700 nm approximately. These optical properties are suitable for performing a high light-harvesting ability in a DSSC device. The absorbance signal for ZnO NWs is mainly located in the UV region, which is due to the high bandgap of ZnO. However, the signal intensity was found to be decreased by decreasing the AR (increasing the diameter) in this region. This result supports *d* = 30 nm as the best possible photoelectrode for harvesting the incident radiation due to the high surface area and density distribution. The inset of Figure 7 displays a magnification between 450 and 700 nm for ZnO NWs and N719 dye. The bands at 513, 559, 566, 577, and 610 nm are associated with the surface plasmon resonance (SPR) effect due to the optical properties of Au nanoparticles located on the tip of ZnO NWs. The band positions presented a blue shift and an increase in the FWHM with an increase in the size of Au diameter (Figure 4). The surface plasmon resonance (SPR) effect is the combined oscillation of unbound electrons of metallic nanoparticles, energized by the incident lights electromagnetic field. When the electrons of nanoparticles are restricted in three dimensions, the electron oscillations stimulate an electric field around the nanoparticle, which can be stronger than that generated by incident radiation (Lou et al., 2013). For DSSC technology, SPR can be utilized to boost the absorption ability of the incident radiation by the photoelectrode because Au nanoparticles produce a light scattering process of the incident radiation when both the incident radiation and SPR wavelength are close to each other. In this work, the absorption peaks located between 513 and 577 nm are adjacent to the absorption band of N719 dye in 525 nm, then the dispersed light due to the SPR effect can be absorbed by the N719 dye (Bora et al., 2013; Thankappan et al., 2015; Lu et al., 2016).

**Figure 8:**
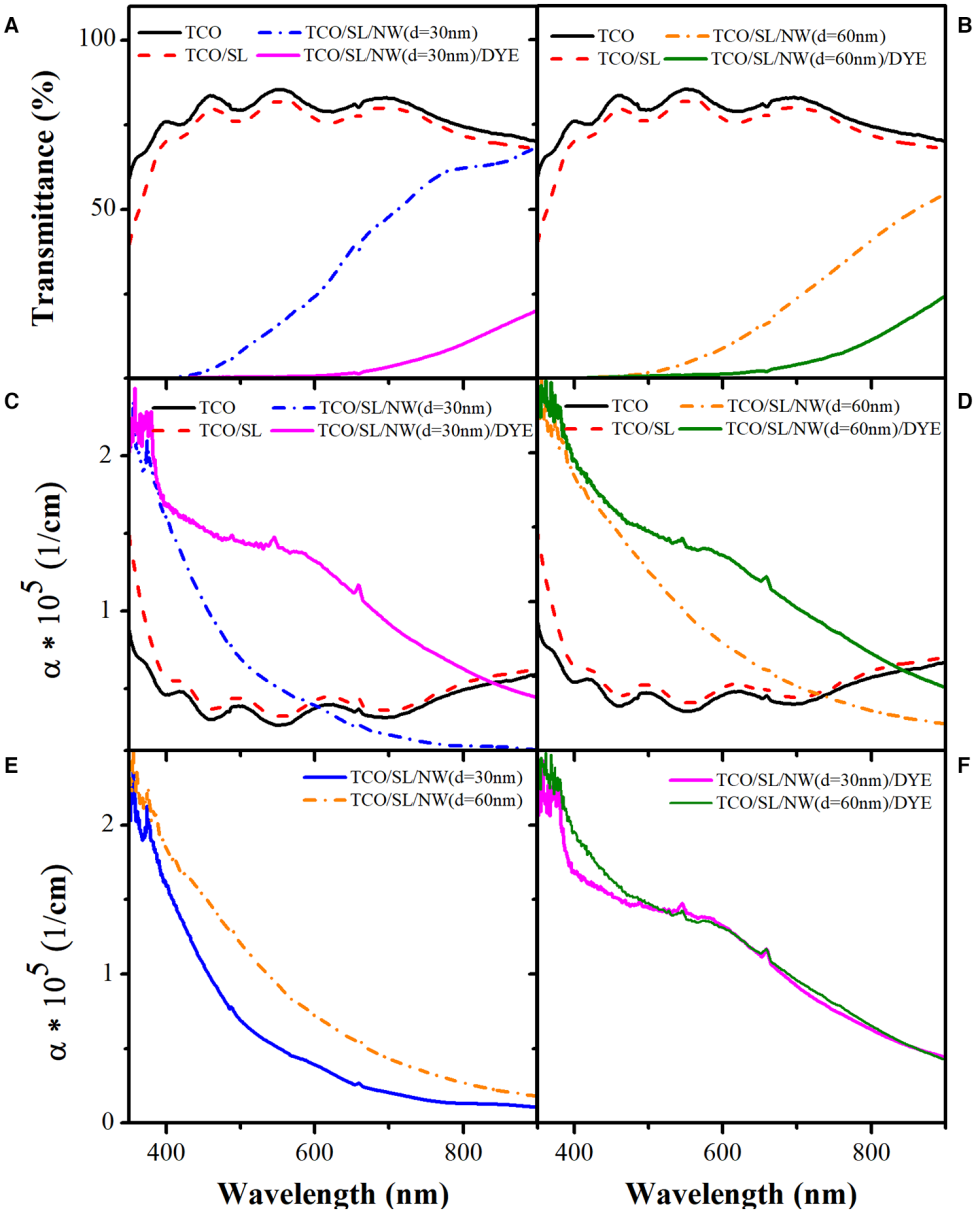
**(A,B)** Transmittance and **(C,D)** absorption coefficient of NW (*d* = 30 nm) and NW (*d* = 60 nm) photoelectrodes, respectively. **(E,F)** Comparison of the absorption coefficient of the photoelectrodes before and after sensitization with N719 dye.

The sensitized photoelectrode prototype is composed of three layers: transparent conductive oxide (TCO), a seed layer (SL), and ZnO NWs. Figures 8A,C show the change in transmittance and absorption coefficient of the NW (*d* = 30 nm) photoelectrode prototype after the sensitization process with N719 dye. As can be seen from Figure 8A, a high transmittance is obtained when TCO is coated over the quartz substrate, and further, it decreases when SL is added. Subsequently, the transmittance signal drops when ZnO NWs are grown by VLS, followed by sensitization with N719 dye. The resulted decrease in transmittance is due to the optical properties of the ZnO NWs, the plasmonic properties of Au nanoparticles, and the absorption properties of N719 dye.

Figure 8C shows the absorption coefficient (α) of the NW (*d* = 30 nm) photoelectrode prototype, which presents a variation corresponding to deposited layers. For TCO and TCO/SL layers, α oscillate along wavelength, whereas for TCO/SL/NW (*d* = 30 nm), α decreases with increasing the wavelength. However, when N719 dye is incorporated as a sensitizer, α increases in the visible region for both *d* = 30 and 60 nm, respectively. A similar trend can be observed for NW (*d* = 60 nm), as shown in Figures 8B,D. This study is carried out to see the influence of the diameter on the absorption properties of two different NWs. Figures 8E,F display α comparison for photoelectrode with NW (*d* = 30 nm) and NW (*d* = 60 nm) before and after the sensitization process. α for both samples decreased with wavelength and increased when the AR decreased. However, after the sensitization process with N719 dye (Figure 8F) α increased in the visible region for both of the electrodes irrespective of the diameter of NWs. A slight difference can be observed in the UV region due to variation in the AR of the samples; however, in the visible region, absorption is due to the combination of both N719 dye and ZnO NWs.

From the previous results and discussions, it can be inferred that the NWs with *d* = 30 nm is one of the best candidates (best crystalline, least defect sites, better orientation, the appropriate density of NWs, and minimal recombination losses) for their use as a possible photoelectrode (anode) in DSSC or other organic solar cells. The high density of NWs and the high aspect ratio can improve the NW surface sites for appropriate functionalizing with the dye. Consequently, an increase in absorption in the dye, generates a greater number of electrons, which will be subsequently transferred to the conduction band (CB) of the ZnO NWs. Due to the high crystalline quality and low-recombination process (*d* = 30 nm), most of the electrons will be transferred to the ETL layer (without loss), which could improve the electrical properties of the DSSC device, such as short circuit current and open-circuit voltage.

Finally, this work can guide the optimal design of photoelectrodes (anode) for the DSSC applications and other organic solar cells.

## Conclusions

ZnO NW photoelectrodes prototypes with a different aspect ratio (AR) were grown by the VLS process. The results show ZnO NWs grow with polycrystalline features and (002) as a preferential plane. The NW dimensions can be well-controlled by the thickness of the Au catalyst, resulting in a high AR of 100; moreover, the NWs presented high-ordered orientation and vertical alignment. The absorbance of the samples introduced a dependence from the AR of NWs and SPR of Au nanoparticles. The NW (*d* = 30 nm) showed better structural and morphological properties, and its optical properties were further improved by N719 dye addition. The obtained results suggest our ZnO NW photoelectrodes prototype (anode) as a potential candidate for their use in optoelectronic applications.

## Data Availability Statement

The original contributions generated for the study are included in the article/supplementary materials, further inquiries can be directed to the corresponding author/s.

## Author Contributions

All authors contributed a direct and intellectual way to the work. KP-C: investigation and writing-original manuscript. AD and GS: supervision, conceptualization, writing-review and editing, and project administration. AM, MB, and MG-S: supervision and review and editing. FG: revision and partial funding. All authors contributed to the discussion and revision of the manuscript.

## Conflict of Interest

The authors declare that the research was conducted in the absence of any commercial or financial relationships that could be construed as a potential conflict of interest.

## References

[B1] Abdin Z. Alim M. A. Saidur R. Islam M. R. Rashmi W. Mekhilef S. (2013). Solar energy harvesting with the application of nanotechnology. Renew. Sustain. Energy Rev. 26, 837–852. 10.1016/j.rser.2013.06.023

[B2] Bedia A. Bedia F. Z. Aillerie M. Maloufi N. Benyoucef B. (2015). Morphological and optical properties of ZnO thin films prepared by spray pyrolysis on glass substrates at various temperatures for integration in solar cell. Energy Procedia 74, 529–538. 10.1016/j.egypro.2015.07.740

[B3] Bisquert J. Cahen D. Hodes G. Rühle S. Zaban A. (2004). Physical chemical principles of photovoltaic conversion with nanoparticulate, mesoporous dye-sensitized solar cells. J. Phys. Chem. B 108, 8106–8118. 10.1021/jp0359283

[B4] Bora T. Kyaw H. H. Dutta J. (2013). Plasmon resonance enhanced zinc oxide photoelectrodes for improvement in performance of dye sensitized solar cells. Mater. Sci. Forum 771, 91–101. 10.4028/www.scientific.net/MSF.771.91

[B5] Boschloo G. (2019). Improving the performance of dye-sensitized solar cells. Front. Chem. 7:77 10.3389/fchem.2019.00077 30838200 PMC6382682

[B6] Canto-Aguilar E. J. Rodríguez-Pérez M. García-Rodríguez R. Lizama-Tzec F. I. De Denko A. T. Osterloh F. E. (2017). ZnO-based dye-sensitized solar cells: effects of redox couple and dye aggregation. Electrochim. Acta 258, 396–404. 10.1016/j.electacta.2017.11.075

[B7] Cui J. (2012). Zinc oxide nanowires. Mater. Charact. 64, 43–52. 10.1016/j.matchar.2011.11.017

[B8] Freitag M. Teuscher J. Saygili Y. Zhang X. Giordano F. Liska P. (2017). Dye-sensitized solar cells for efficient power generation under ambient lighting. Nat. Photonics 11, 372–378. 10.1038/nphoton.2017.60

[B9] Galdamez A. Serrano A. Santana G. Arjona N. Arriaga L. G. Tapia Ramirez J. (2019). DNA probe functionalization on different morphologies of ZnO/Au nanowire for bio-sensing applications. Mater. Lett. 235, 250–253. 10.1016/j.matlet.2018.10.026

[B10] Galdámez-Martínez A. Bai Y. Santana G. Sprick R. S. Dutt A. (2020a). Photocatalytic hydrogen production performance of 1-D ZnO nanostructures: role of structural properties. Int. J. Hydrog. Energy 45, 31942–31951. 10.1016/j.ijhydene.2020.08.247

[B11] Galdámez-Martinez A. Santana G. Güell F. Martínez-Alanis P. R. Dutt A. (2020b). Photoluminescence of zno nanowires: a review. Nanomaterials 10:E857. 10.3390/nano10050857 32365564 PMC7712396

[B12] Gao Y. F. Nagai M. Chang T. C. Shyue J. J. (2007) Solution derived ZnO nanowire array film as photoelectrode in dye sensitized solar cells. Cryst. Growth Des. 7, 2467–2471. 10.1021/cg060934k20407141

[B13] Gong J. Liang J. Sumathy K. (2012). Review on dye-sensitized solar cells (DSSCs): fundamental concepts and novel materials. Renew. Sustain. Energy Rev. 16, 5848–5860. 10.1016/j.rser.2012.04.044

[B14] Gong J. Sumathy K. Qiao Q. Zhou Z. (2017). Review on dye-sensitized solar cells (DSSCs): advanced techniques and research trends. Renew. Sustain. Energy Rev. 68, 234–246. 10.1016/j.rser.2016.09.097

[B15] Grätzel M. (2005). Solar energy conversion by dye-sensitized photovoltaic cells. Inorg. Chem. 44, 6841–6851. 10.1021/ic0508371 16180840

[B16] Greenaway A. L. Boucher J. W. Oener S. Z. Funch C. J. Boettcher S. W. (2017). Low-cost approaches to III-V semiconductor growth for photovoltaic applications. ACS Energy Lett. 2, 2270–2282. 10.1021/acsenergylett.7b00633

[B17] Güell F. Martínez-Alanis P. R. (2019). Tailoring the green, yellow and red defect emission bands in ZnO nanowires via the growth parameters. J. Lumin. 210, 128–134. 10.1016/j.jlumin.2019.02.017

[B18] Güell F. Martínez-Alanis P. R. Roso S. Salas-pérez C. I. García-sánchez M. F. Santana G. (2016). Plasma versus thermal annealing for the Au-catalyst growth of ZnO nanocones and nanowires on Al-doped ZnO buffer layers. Mater. Res. Express 3, 1–11. 10.1088/2053-1591/3/6/065013

[B19] Hagfeldt A. Boschloo G. Sun L. Kloo L. Pettersson H. (2010). Dye-sensitized solar cells. Chem. Rev. 110, 6595–6663. 10.1021/cr900356p 20831177

[B20] Hong W. K. Jo G. Kwon S. S. Song S. Lee T. (2008). Electrical properties of surface-tailored ZnO nanowire field-effect transistors. IEEE Trans. Electron Devices 55, 3020–3029. 10.1109/TED.2008.2005156

[B21] Hsu H. C. Cheng C. S. Chang C. C. Yang S. Chang C. S. Hsieh W. F. (2005). Orientation-enhanced growth and optical properties of ZnO nanowires grown on porous silicon substrates. Nanotechnology 16, 297–301. 10.1088/0957-4484/16/2/021 21727439

[B22] Juang S. S. Y. Lin P. Y. Lin Y. C. Chen Y. S. Shen P. S. Guo Y. L.. (2019). Energy harvesting under dim-light condition with dye-sensitized and perovskite solar cells. Front. Chem. 7, 1–9. 10.3389/fchem.2019.00209 31024895 PMC6465951

[B23] Jung H. S. Lee J.-K. (2013). Dye sensitized solar cells for economically viable photovoltaic systems. J. Phys. Chem. Lett. 4, 1682–1693. 10.1021/jz400112n 26282979

[B24] Kang S. W. Mohanta S. K. Kim Y. Y. Cho H. K. (2008). Realization of vertically well-aligned ZnO:Ga nanorods by magnetron sputtering and their field emission behavior. Cryst. Growth Des. 8, 1458–1460. 10.1021/cg701216f

[B25] Karst N. Rey G. Doisneau B. Roussel H. Deshayes R. Consonni V. (2011). Fabrication and characterization of a composite ZnO semiconductor as electron transporting layer in dye-sensitized solar cells. Mater. Sci. Eng. B Solid State Mater. Adv. Technol. 176, 653–659. 10.1016/j.mseb.2011.02.009

[B26] Lee T.-H. Sue H.-J. Cheng X. (2011). ZnO and conjugated polymer bulk heterojunction solar cells containing ZnO nanorod photoanode. Nanotechnology 22:285401. 10.1088/0957-4484/22/28/285401 21625040

[B27] Lou Y. Yuan S. Zhao Y. Hu P. Wang Z. Zhang M.. (2013). Molecular-scale interface engineering of metal nanoparticles for plasmon-enhanced dye sensitized solar cells. Dalt. Trans. 42, 5330–5337. 10.1039/c3dt32741h 23407603

[B28] Lu M. Y. Tsai C. Y. Chen H. A. Liang Y. T. Chen K. P. Gradečak S. (2016). Plasmonic enhancement of Au nanoparticle-embedded single-crystalline ZnO nanowire dye-sensitized solar cells. Nano Energy 20, 264–271. 10.1016/j.nanoen.2015.12.026

[B29] Meng L. Chen H. Li C. dos Santos M. P. (2014). Growth of the [110] oriented TiO 2 nanorods on ITO substrates by sputtering technique for dye-sensitized solar cells. Front. Mater. 1:14 10.3389/fmats.2014.00014

[B30] O'Regan B. Grätzel M. (1991). A low-cost, high-efficiency solar-cell based on dye-sensitized colloidal TiO_2_ films. Nature 353, 737–740. 10.1038/353737a0

[B31] Parisi M. L. Maranghi S. Basosi R. (2014). The evolution of the dye sensitized solar cells from Grätzel prototype to up-scaled solar applications: a life cycle assessment approach. Renew. Sustain. Energy Rev. 39, 124–138. 10.1016/j.rser.2014.07.079

[B32] Pathak N. K. (2020). Plasmonic nanostructures for energy application. Front. Mech. Eng. 6, 1–5. 10.3389/fmech.2020.00053

[B33] Polyakov B. Kuzmin A. Smits K. Zideluns J. Butanovs E. Butikova J. (2016). Unexpected epitaxial growth of a few WS2 Layers on {1100} facets of ZnO nanowires. J. Phys. Chem. C 120, 21451–21459. 10.1021/acs.jpcc.6b06139

[B34] Portillo-Cortez K. Martínez A. Dutt A. Santana G. (2019). N719 Derivatives for application in a dye-sensitized solar cell (DSSC): a theoretical study. J. Phys. Chem. A 123, 10930–10939. 10.1021/acs.jpca.9b09024 31799849

[B35] Sakai N. Miyasaka T. Murakami T. N. (2013). Efficiency enhancement of ZnO-based dye-sensitized solar cells by low-temperature TiCl4 treatment and dye optimization. J. Phys. Chem. C 117, 10949–10956. 10.1021/jp401106u

[B36] Saleem M. Farooq W. A. Khan M. I. Akhtar M. N. Rehman S. U. Ahmad N. (2019) Effect of ZnO nanoparticles coating layers on top of ZnO nanowires for morphological, optical, photovoltaic properties of dye-sensitized solar cells. Micromachines 10, 1–11. 10.3390/mi10120819. 31779196 PMC6953122

[B37] Scalia A. Varzi A. Lamberti A. Jacob T. Passerini S. (2018). Portable high voltage integrated harvesting-storage device employing dye-sensitized solar module and all-solid-state electrochemical double layer capacitor. Front. Chem. 6:443. 10.3389/fchem.2018.00443 30320074 PMC6167942

[B38] Serrano A. Arana A. Galdámez A. Dutt A. Monroy B. M. Güell F. (2017). Effect of the seed layer on the growth and orientation of the ZnO nanowires: consequence on structural and optical properties. Vacuum 146, 509–516. 10.1016/j.vacuum.2017.03.010

[B39] Simon H. Krekeler T. Schaan G. Mader W. (2013). Metal-seeded growth mechanism of ZnO nanowires. Cryst. Growth Des. 13, 572–580. 10.1021/cg301640v

[B40] Skandalos N. Karamanis D. (2015). PV glazing technologies. Renew. Sustain. Energy Rev. 49, 306–322. 10.1016/j.rser.2015.04.145

[B41] Song H. S. Zhang W. J. Tang Y. B. He Z. B. Yuan G. D. Fan X.. (2010). Field electron emission of ZnO nanowire pyramidal bundle arrays. J. Nanosci. Nanotechnol. 10, 2360–2365. 10.1166/jnn.2010.1916 20355434

[B42] Thankappan A. Divya S. Augustine A. K. Girijavallaban C. P. Radhakrishnan P. Thomas S. (2015). Highly efficient betanin dye based ZnO and ZnO/Au Schottky barrier solar cell. Thin Solid Films 583, 102–107. 10.1016/j.tsf.2015.03.052

[B43] Udom I. Ram M. K. Stefanakos E. K. Hepp A. F. Goswami D. Y. (2013). One dimensional-ZnO nanostructures: synthesis, properties and environmental applications. Mater. Sci. Semicond. Process. 16, 2070–2083. 10.1016/j.mssp.2013.06.017

[B44] Wang Q. Ito S. Graetzel M. Fabregat-Santiago F. Mora-Sero I. Bisquert J.. (2006). Characteristics of high efficiency dye-sensitized solar cells. J. Phys. Chem. B 110, 25210–25221. 10.1021/jp064256o 17165965

[B45] Warnan J. Guerin V. M. Anne F. B. Pellegrin Y. Blart E. Jacquemin D. (2013). Ruthenium sensitizer functionalized by acetylacetone anchoring groups for dye-sensitized solar cells. J. Phys. Chem. C 117, 8652–8660. 10.1021/jp402608u

[B46] Wijeratne K. Bandara J. (2014). Aspect-ratio dependent electron transport and recombination in dye-sensitized solar cells fabricated with one-dimensional ZnO nanostructures. Electrochim. Acta 148, 302–309. 10.1016/j.electacta.2014.10.046

[B47] Yang H. J. Lim S. C. He S. Y. Tuan H. Y. (2015). Ultralong mesoporous ZnO nanowires grown via room temperature self-assembly of ZnO nanoparticles for enhanced reversible storage in lithium ion batteries. RSC Adv. 5, 33392–33399. 10.1039/C5RA01423A

[B48] Yang L. L. Yang J. H. Wang D. D. Zhang Y. J. Wang Y. X. Liu H. (2008). Photoluminescence and Raman analysis of ZnO nanowires deposited on Si(1 0 0) via vapor-liquid-solid process. Phys. E Low Dimens. Syst. Nanostruct. 40, 920–923. 10.1016/j.physe.2007.11.025

[B49] Ye M. Wen X. Wang M. Iocozzia J. Zhang N. Lin C. (2015). Recent advances in dye-sensitized solar cells: from photoanodes, sensitizers and electrolytes to counter electrodes. Mater. Today 18, 155–162. 10.1016/j.mattod.2014.09.001

[B50] Zhang Y. Ram M. K. Stefanakos E. K. Goswami D. Y. (2012). Synthesis, characterization, and applications of ZnO nanowires. J. Nanomater. 2012:624520 10.1155/2012/624520

